# Research progress on the prevention and treatment of exercise-induced fatigue with ginseng and relevant formulas

**DOI:** 10.3389/fphar.2026.1764382

**Published:** 2026-04-02

**Authors:** Li Yi, Shangjin Song, Xiaoxia Qi, Hua Jing, Wei Gu, Yi Ruan

**Affiliations:** 1 Naval Medical Center, Shanghai, China; 2 Department of Traditional Chinese Medicine, Naval Medical University, Shanghai, China

**Keywords:** clinical application, exercise-induced fatigue, ginseng, ginseng-containing formulas, mechanism, research progress

## Abstract

In recent years, numerous studies have confirmed that ginseng and ginseng-containing formulas exert significant preventive and therapeutic effects on exercise-induced fatigue (EIF). Their anti-fatigue effects are mainly mediated by regulating neurotransmitter release, modulating cytokine receptor binding, and interfering with cell adhesion molecule interactions. This review systematically analyzes and discusses the research progress of ginseng and ginseng-containing formulas in EIF management from the perspectives of formula composition, pharmacological mechanism, and clinical application, with the aim of providing a theoretical basis for syndrome differentiation-based treatment and precise clinical medication of EIF.

## 1 Introduction

Exercise-induced fatigue (EIF) refers to a physiological state where the body’s exercise capacity declines and physiological functions are altered, caused by continuous energy consumption and metabolic product accumulation during prolonged or high-intensity exercise (Margaritelis et al., 2020). Its occurrence and progression are closely linked to the interaction of multiple pathological mechanisms, including energy metabolism imbalance, muscle function impairment, central nervous system regulation disorder, and excessive oxidative stress response. From a physiological perspective, EIF is not only the basis for the body to adapt to various changes during exercise, but also an important physiological protective mechanism (Gao and Duan, 2024). However, if the fatigue state accumulates continuously and the body fails to recover in time, physiological fatigue may transform into pathological fatigue, which can lead to overtraining syndrome, damage the body, and seriously impair executive function and cognitive ability (Ma et al., 2018). In traditional Chinese medicine (TCM), EIF is classified into the categories of “xulao” (consumptive exhaustion) and “laojian” (fatigue impairment), with the core pathogenesis being “lao ze hai qi” (fatigue consumes vital qi). This further causes decline or disorder of visceral functions, accompanied by deficiency of yin-fluid and blood, manifesting as a temporary state of internal injury and consumptive fatigue (Wang and Zhang, 2015).

The core pathological features of EIF (energy metabolism disorder, oxidative stress, neuroendocrine disturbance) are highly consistent with the efficacy targets of ginseng (tonifying qi and nourishing yin, reinforcing healthy qi), making it a key botanical drug for anti-fatigue intervention.

Ginseng is a core qi-tonifying botanical drug in TCM, well-known for reinforcing healthy qi and invigorating vital energy. It is widely used in clinical practice across internal medicine, surgery, gynecology, pediatrics, and orthopedics, and is a core component of numerous classic TCM formulas. For EIF, ginseng-containing formulas have shown significant therapeutic effects: balancing energy metabolism, regulating the central nervous system, enhancing muscle function, and alleviating oxidative stress. Its therapeutic value lies in the comprehensive conditioning of the body and promotion of qi and blood production, thereby reducing the severity of EIF (Jin et al., 2020).

Compared with synthetic anti-fatigue drugs, ginseng and ginseng-containing formulas have three key advantages: (1) They are safe and well-tolerated, supported by centuries of traditional use and modern research (Committee National Pharmacopoeia, 2020; Park et al., 2018; Lee et al., 2012; Kim et al., 2015; Jeon et al., 2024); (2) Their active compounds, like ginsenosides, target multiple pathways to reduce exercise-induced fatigue (Jin et al., 2020; Lu et al., 2021); (3) They balance strengthening the body and removing fatigue causes, aligning with traditional Chinese medicine principles for better results than symptom-only treatments (Hai-Bo and Shi-Ming, 2017). Unlike existing reviews, the present work is the first to integrates the correlation among the TCM syndrome differentiation types of exercise-induced fatigue, the modern medical molecular mechanisms, and the clinical application of ginseng-containing formulas, and systematically covers both classic ginseng-containing formulas and modern empirical formulas, providing a more comprehensive reference for clinical precise medication. This article will systematically discuss the etiology and pathogenesis of exercise-induced fatigue, review the research progress of ginseng and ginseng-containing formulas in preventing and treating exercise-induced fatigue, summarize their core characteristics, and provide references for subsequent clinical practice and basic research (Zhang, 1997).

## 2 Literature search and screening methods

**FIGURE 1:**
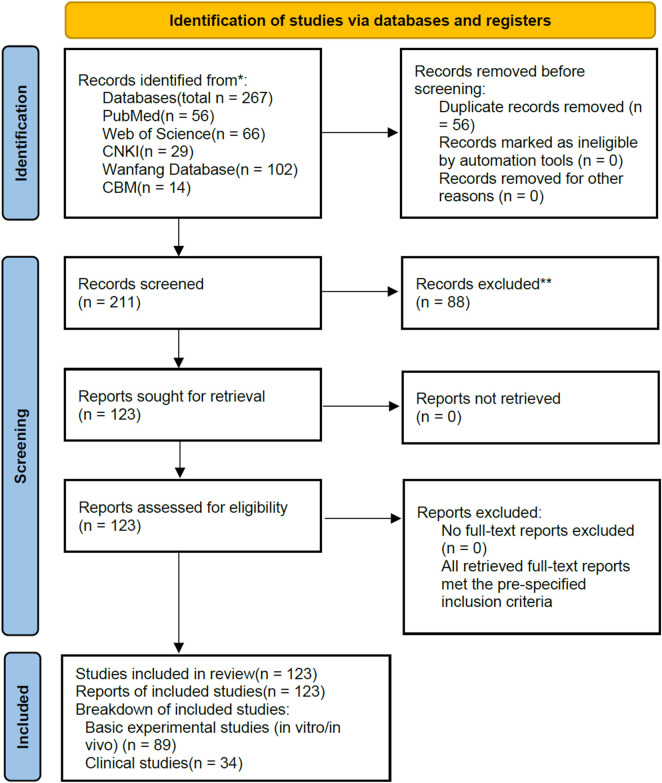
PRISMA 2020 flow diagram of study selection. Note: *Databases include PubMed, Web of Science, CNKI, Wanfang Database, and CBM. The retrieval time frame is from June 2015 to June 2025, and a combination of subject terms and free words was used for comprehensive retrieval. **Reason for exclusion: Irrelevant studies inconsistent with the research topic. Title and abstract screening were completed by independent manual review, no automation tools were used.

This review adhered to systematic review reporting guidelines for literature retrieval and screening. Relevant studies on ginseng and ginseng-containing formulas for exercise-induced fatigue (EIF) were searched in PubMed, Web of Science, CNKI, Wanfang Database and CBM, with the retrieval time range from June 2015 to June 2025. English search terms included “Panax ginseng”, “Ginseng”, “Ginsenosides”, “Ginseng polysaccharides”, “Ginseng-containing formulas”, “Exercise-induced fatigue”, “Physical fatigue”, “Exercise-related fatigue”, “Fatigue relief”, “Fatigue improvement”, “Mechanism”, “Molecular mechanism”, “Signaling pathway” and “Clinical application”; Chinese search terms were Panax ginseng (人参), Ginsenosides (人参皂苷), Ginseng polysaccharides (人参多糖), Ginseng-containing formulas (含人参方), Exercise-induced fatigue (运动性疲劳), Physical fatigue (体力疲劳), Exercise-related fatigue (运动相关疲劳), Fatigue relief (缓解疲劳), Fatigue improvement (改善疲劳), Mechanism (作用机制), Molecular mechanism (分子机制), Signaling pathway (信号通路) and Clinical application (临床应用),using a combination of subject terms and free words for comprehensive retrieval. Inclusion criteria: original basic experimental studies (*in vitro*/*in vivo*),clinical studies, systematic reviews/meta-analyses with complete data, and Chinese/English literatures with extractable key data. Exclusion criteria: duplicate publications, conference abstracts, dissertations, irrelevant studies, and those with flawed research design. A total of 267 literatures were initially retrieved, with 56 duplicates removed and 88 irrelevant ones excluded by title/abstract screening. Finally, 123 eligible literatures were included (89 basic experimental studies and 34 clinical studies), covering both classical and contemporary ginseng-containing formulas, as well as the anti-EIF mechanisms of ginsenosides, polysaccharides and other active components (Figure 1).

## 3 Understanding of exercise-induced fatigue in traditional Chinese and western medicine

### 3.1 Traditional Chinese medicine’s understanding of exercise-induced fatigue

In traditional Chinese medicine, the earliest literature record of the term “fatigue” can be found in the *“Huangdi Neijing” (Yellow Emperor’s Inner Classic)*, and there are also relevant records in *“Jinkui Yaolue: Blood Stasis and Exhaustion”*: “Those who are honored and have weak bones but strong muscles, if they are overworked and sweat, will suffer from exhaustion.” This reflects the early understanding of fatigue-related disorders in traditional Chinese medicine. The *“Huangdi Neijing”* points out that the occurrence of fatigue is related to both internal and external factors, with internal injury (especially overwork) being the main cause. Moreover, the concept of *“Five Labors and Seven Injuries”* mentioned in the book summarizes that “prolonged visual strain depletes blood, prolonged lying depletes qi, prolonged sitting depletes flesh, prolonged standing depletes bones, and prolonged walking depletes tendons,” which are all manifestations of physical fatigue (Su, 1994).

Regarding the mechanism of exercise-induced fatigue, *“Jingyue Quanshu: Xu Shi”* (Zhang, 1991) states: “If one does not know their limits and forces themselves to do things beyond their capacity, all such actions can cause damage.” This clearly indicates that excessive fatigue occurs when the body exceeds its tolerance level, leading to excessive consumption. Additionally, “*San Yin Ji Yi Bing Zheng Fang Lun: San Yin Lun”* (Chen, 1991) mentions: “Overeating, overdrinking, excessive shouting, overthinking, and overexertion are internal causes,” *and “Jinkui Yuhuan Yaolue Jiwen”* states: “Injury to qi will affect essence,” as well as *“Danxi Xinfa: Deafness”* (Zhu, 1999) which says: “Excessive labor and sexual indulgence deplete blood and qi.” These all suggest that fatigue is not merely a physical depletion but also involves the deficiency of essence, qi, blood, body fluids, and damage to visceral functions. According to *“Suwen: Shi Congrong Lun,”* “Liver deficiency, kidney deficiency, and spleen deficiency all cause heaviness and distress in the body,” indicating that although the pathogenesis of fatigue may involve all five viscera, the core pathogenesis is often related to the dysfunction of the liver, spleen, and kidney. In terms of pathogenic factors, the *“Suwen: Discussion on Pain”* proposes that “excessive labor leads to qi depletion”, and the *“Suwen: Discussion on Regulating the Meridians”* states that “excessive fatigue leads to a decline in physical condition and qi”, both clearly listing fatigue as an important pathogenic factor (Zhou, 2007). Modern TCM research has a more in-depth and detailed understanding and classification of fatigue. Based on the TCM syndrome differentiation theory of fatigue, some practitioners have classified exercise-induced fatigue into three types with a total of 12 common syndromes, and have formulated corresponding diagnostic criteria for these 12 syndromes to better understand the etiology and pathogenesis and provide more targeted treatment ideas for clinical practice (Rong and Zhang, 2017). In the treatment of fatigue, TCM takes “harmony” as the core principle, and through methods such as strengthening the body and eliminating pathogenic factors, tonifying the middle and benefiting qi, lifting yang and supporting the sinking, and harmonizing the spleen and stomach, it improves the patient’s fatigue symptoms, promotes smooth qi circulation, harmonizes the internal and external organs, and restores the balance of yin and yang, ultimately helping the patient regain normal physical strength.

### 3.2 Mechanism of exercise-induced fatigue

The mechanism of exercise-induced fatigue is complex and not yet fully understood. Its occurrence involves the coordinated stress response and metabolic regulation disorder of the central nervous system, skeletal muscles, and multiple tissues and organs, which can be divided into two major categories: central fatigue and peripheral fatigue (Gao and Duan, 2024).

#### 3.2.1 Classic mechanisms

Exercise-induced fatigue (EIF) stems from multiple interconnected pathological mechanisms, which ginseng and ginseng-based formulas aim to address. Classic theories explain EIF through five key perspectives. (1) Environmental homeostasis imbalance theory: High-intensity exercise disrupts the body’s balance, impairing cell function and reducing exercise capacity (Grauffel et al., 2019); (2) Free radical theory: Intense exercise increases free radicals, causing oxidative damage and fatigue (Meng and Su, 2024), Abnormal Nrf2 antioxidant pathways worsen this (Gallego-Selles et al., 2020). (3) Metabolic product accumulation theory: Anaerobic exercise leads to buildup of lactic acid and ammonia, causing acid-base imbalance and lower muscle efficiency (Sundberg and Fitts, 2019); Ammonia also disrupts brain chemicals, inducing central fatigue (Holeček, 2022). (4) Energy depletion theory: Exercise relies on energy systems like phosphate and glycolysis (Hargreaves et al., 2018; Koh et al., 2022; Hargreaves and Spriet, 2020), Over-exercise depletes energy stores, triggering fatigue (Hargreaves and Spriet, 2020). (5) Protective inhibition theory: Long-term training affects neurotransmitters like DA and 5-HT, leading to brain inhibition and declining performance (Liu Y. et al., 2019; Tornero-Aguilera et al., 2022).

#### 3.2.2 Molecular mechanisms

Important breakthroughs have been made in the study of molecular mechanisms of EIF in recent years, which further reveal the core regulatory pathways of fatigue occurrence and progression.

##### 3.2.2.1 Neuroendocrine regulation

Neuroendocrine remodeling is a key trigger of central fatigue. The Brain-Derived Neurotrophic Factor (BDNF)/TrkB.T1 pathway in ventromedial hypothalamus (VMH) astrocytes can regulate synaptic glutamate uptake and energy homeostasis, and dysfunction of this pathway can accelerate the onset of fatigue (Ameroso et al., 2022); In addition, dysfunction of the leptin-BDNF pathway can lead to lipid metabolism disorders and impair exercise capacity (Wang et al., 2020).

##### 3.2.2.2 Epigenetic modulation

Abnormal epigenetic regulation is widely involved in the pathological process of fatigue. Adenosine 5′-monophosphate-activated protein kinase (AMPK), the core molecule of energy homeostasis, can promote mitochondrial biogenesis by activating Peroxisome Proliferator-Activated Receptor γ Coactivator 1α (PGC-1α). Meanwhile, Sirtuin 6 (SIRT6) maintains redox balance by regulating Nrf2 signaling. Abnormal function of these two key molecules can exacerbate systemic energy metabolism disorders in EIF (Pan et al., 2016).

##### 3.2.2.3 Metabolic pathway imbalance

The imbalance of the Tryptophan-Kynurenine (TRP-KYN) metabolic pathway plays a critical role in EIF progression. Overtraining can induce the release of pro-inflammatory factors, leading to the accumulation of neurotoxic metabolites. In contrast, regular exercise can promote the production of neuroprotective substances through the Peroxisome Proliferator-Activated Receptor γ (PPARγ) pathway, and this imbalance is a key link in the occurrence and persistence of fatigue (Martin et al., 2020). In addition, dysfunction of the muscle-organ crosstalk mechanism mediated by muscle-secreted myokines such as irisin and adiponectin can also promote the development of fatigue (Severinsen and Pedersen, 2020).

The occurrence of EIF is the result of a vicious cycle formed by the crosstalk of multiple interconnected mechanisms: energy metabolism disorder induced by prolonged or high-intensity exercise directly triggers excessive oxidative stress (Meng and Su, 2024), which further disrupts neuroendocrine homeostasis (e.g., dysfunction of the BDNF/TrkB.T1 pathway and hypothalamic-pituitary-adrenal (HPA) axis) (Ameroso et al., 2022), and ultimately leads to the simultaneous onset of central and peripheral fatigue (Gao and Duan, 2024). Panax ginseng and its core active metabolites (e.g., ginsenosides Rg1, Rg3, Rb1) can block this vicious cycle at multiple key nodes: it regulates energy metabolism via the PI3K/Akt/mTOR and AMPK/PGC-1α pathways (Peng et al., 2024), alleviates oxidative stress by enhancing the activity of antioxidant enzymes (superoxide dismutase (SOD), catalase (CAT)) and inhibiting malondialdehyde (MDA) production (Song, 2017), and corrects neuroendocrine disturbance by balancing the levels of central neurotransmitters (5-HT, GABA, DA) (Peng et al., 2024).

## 4 The mechanism of ginseng (roots of *Panax ginseng* C. A. Mey. [Araliaceae]) in alleviating exercise-induced fatigue

### 4.1 Anti-fatigue effects of core active metabolites

In recent years, numerous studies have confirmed the significant anti-EIF effects of ginseng (Zhu et al., 2022). The core anti-fatigue active metabolites of ginseng mainly include ginsenosides, ginseng polysaccharides, and ginseng proteins. Among them, ginsenosides have become the focus of academic research due to their clear and well-verified mechanism of action. To date, 123 ginsenoside monomers have been discovered and reported (Song, 2017). Among ginsenoside monomers, the anti-fatigue effect of ginsenoside Rg1 has been fully verified. Studies using exhaustive fatigue models have found that Rg1 can significantly increase the activity of antioxidant enzymes including superoxide dismutase (SOD) and catalase (CAT) in exercise-trained rats, while reducing the levels of malondialdehyde (MDA) and carbonylated proteins (CPs), thereby alleviating oxidative stress damage and attenuating exercise-induced fatigue (Fan et al., 2016). In addition, this metabolite can regulate the levels of central neurotransmitters including 5-hydroxytryptamine (5-HT), γ-aminobutyric acid (GABA), and dopamine (DA) in the hypothalamus, correct the disorder of central neurotransmitters, and exert an anti-central fatigue effect (Feng et al., 2010). Ginsenoside Rg3 also shows broad-spectrum anti-fatigue activity. On the one hand, it can increase muscle glycogen reserves in fatigued mice, reduce blood urea nitrogen (BUN) levels, and enhance the activity of Na^+^,K^+^-ATPase in muscle tissue to alleviate exercise fatigue. On the other hand, it can increase mitochondrial membrane potential and respiratory chain complex activity in skeletal muscle to improve hypoxia-related fatigue (Zhang H. et al., 2022). It also acts as a SIRT1 agonist to promote mitochondrial biogenesis by activating the SIRT1/PGC-1α pathway, thereby enhancing fatigue resistance in aging organisms (Yang et al., 2018). Ginsenoside Rb1 exerts anti-fatigue effects through multiple pathways. It can not only improve skeletal muscle energy metabolism, increase intracellular ATP content and succinate dehydrogenase (SDH) activity (Zhang Z. et al., 2022), but also reduce the secretion of inflammatory factors by inhibiting the p38MAPK–NF-κB/p65 pathway to alleviate fatigue-related inflammatory responses (Liu et al., 2021). It can also regulate the levels of central neurotransmitters to improve the body’s tolerance to central fatigue (Feng et al., 2010). In addition to ginsenosides, ginseng stem and leaf polysaccharides can effectively scavenge free radicals induced by intense exercise in rats, and increase energy reserves by regulating liver glycogen synthesis, which work together with ginsenosides to exert anti-fatigue effects (Tian, 2015; Lin et al., 2015). Cultured ginseng adventitious root proteins can reduce oxidative damage caused by hydrogen peroxide (H_2_O_2_) intervention, lower intracellular MDA levels, and maintain normal cell function to resist fatigue (Wang et al., 2022).

### 4.2 Regulatory mechanism of molecular signaling pathways

At the molecular level, ginseng extracts exert anti-EIF effects through multi-pathway regulation. First, ginseng extracts can promote glucose uptake and glycogen synthesis by activating the phosphatidylinositol-3-hydroxy kinase (PI3K)/protein kinase B (Akt)/mammalian target of rapamycin (mTOR) signaling pathway, thereby improving energy metabolism and physical recovery in chronically fatigued rats (Zhang et al., 2023; Chen et al., 2024). Second, they can regulate the expression of core target genes in tumor necrosis factor (TNF), interleukin 17 (IL-17) and other inflammatory signaling pathways, further enhancing the anti-fatigue effect by alleviating chronic low-grade inflammation (Liu FX. et al., 2019). In addition, the intestinal microbiota plays a key mediating role in the anti-fatigue effect of ginseng. The polysaccharides and saponins in ginseng water extract can regulate the composition of intestinal microbiota, promote the metabolism of short-chain fatty acids (SCFA) and bile acids, improve energy metabolism disorders and oxidative stress, and exert a comprehensive anti-EIF effect (Sun et al., 2025). Meanwhile, ginseng can regulate immune homeostasis by stimulating B lymphocyte proliferation and enhancing the phagocytic function of macrophages, reversing the decline of immune function related to fatigue (Zhang, 2022). It can also promote nitric oxide (NO) release to improve microcirculation, reduce blood viscosity, and enhance tissue oxygen supply efficiency, providing multiple auxiliary anti-fatigue effects (Lu et al., 2021).

### 4.3 Synergistic effect of active metabolites and gut microbiota mediation

The anti-fatigue effect of ginseng is the result of the combined action of multiple active metabolites, among which ginsenosides and ginseng polysaccharides exert complementary regulatory effects on the body’s anti-fatigue system (Zhu et al., 2022; Tian, 2015). Meanwhile, ginseng can upregulate the abundance of beneficial intestinal bacteria such as Bifidobacterium and *Lactobacillus*, and their metabolic product SCFA can improve EIF-related energy metabolism disorders, thereby mediating the anti-fatigue effect of ginseng (Sun et al., 2025). Animal experiments have shown that the administration dose of ginsenosides, when converted to the human equivalent dose (HED), is basically consistent with the conventional clinical dosage of ginseng, indicating that the existing research results have certain clinical translation feasibility (Lu et al., 2021). Specifically, the administration dose of ginsenoside Rg1 in animal experiments (SD rats) is 50 mg/kg (Feng et al., 2010). Using the body surface area conversion method (rat-to-human conversion factor of 0.16), the HED of Rg1 is 8 mg/kg, which is consistent with the common clinical dosage of ginseng extracts (100–3,000 mg/d) containing ginsenoside Rg1 (Zhu et al., 2022), further verifying the potential of clinical translation.

**FIGURE 2:**
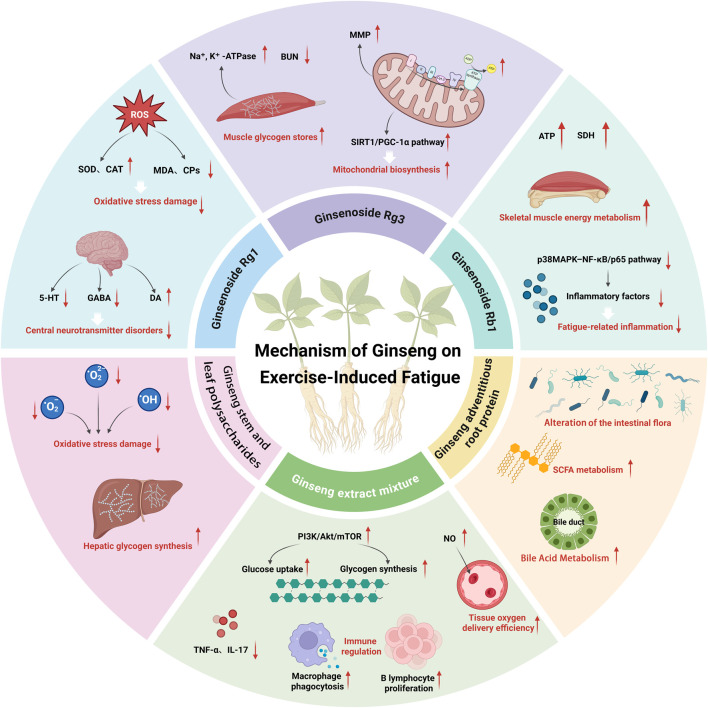
Integrated diagram of the multi-pathway mechanism of Ginseng and Ginseng-containing metabolites/formulas in resisting exercise-induced fatigue. Note: In the figure, the arrow “↑” indicates “activation/promotion” and “↓” indicates “inhibition/reduction”; the core elements are explained as follows: ① Ginsenosides (Rg1, Rg3, Rb1), Ginseng stem and leaf polysaccharides, Ginseng adventitious root proteins; ② Key pathways: SIRT1/PGC-1α, PI3K/Akt/mTOR, p38MAPK-NF-κB/p65, AMPK/PGC-1α, etc.,; ③ Core indicators: Antioxidant-related (SOD Superoxide Dismutase, CAT Catalase, MDA Malondialdehyde, CPs Carbonylated Proteins), energy metabolism-related (Muscle glycogen, Hepatic glycogen, ATP Adenosine Triphosphate, Na^+^,K^+^-ATPase Sodium-Potassium ATPase, SDH Succinate Dehydrogenase), fatigue markers (BUN Blood Urea Nitrogen, LDH Lactate Dehydrogenase), neurotransmitters (5-HT 5-Hydroxytryptamine, GABA γ-Aminobutyric Acid, DA Dopamine), inflammatory factors (TNF-α Tumor Necrosis Factor-α, IL-17 Interleukin-17); ④ Physiological effects: Alleviating oxidative stress damage, regulating energy metabolism homeostasis, inhibiting fatigue-related inflammation, repairing central neurotransmitter disorders, regulating intestinal flora (SCFA Short-Chain Fatty Acids), enhancing immune function (macrophage phagocytosis, B lymphocyte proliferation), and improving tissue oxygen supply efficiency (NO Nitric Oxide). This figure systematically shows the core mechanism network by which ginseng and related metabolites/formulas alleviate exercise-induced fatigue through multi-metabolite combination effects and multi-pathway regulation.

The above studies confirm that ginseng and its extracts can significantly enhance the body’s exercise capacity and fatigue tolerance through multiple pathways, including anti-oxidation, energy metabolism regulation, neural regulation, inflammation inhibition, intestinal microbiota regulation, and immune balance maintenance. Notably, existing studies on the anti-EIF effects of ginseng still have prominent limitations: most basic experiments use young healthy mouse models, which have poor clinical relevance to the actual population with EIF (Zhang H. et al., 2022; Wang et al., 2022); most mechanism studies focus on single metabolites or single pathways, ignoring the combined effects of multiple components and multi-pathway crosstalk (Sun et al., 2025); some studies lack standardized extract concentration limits and clear taxonomic validation of ginseng species (Lu et al., 2021). These deficiencies restrict the clinical translation of existing findings, and call for more rigorous and systematic investigations in future research (Figure 2).

## 5 Ginseng-containing formulas

### 5.1 Buzhong Yiqi Decoction

Core ingredients and classic proportion: Panax ginseng C. A. Mey. (Araliaceae, 10 g), Astragalus membranaceus (Fisch.) Bunge (Fabaceae, 15 g), Atractylodes macrocephala Koidz. (Asteraceae, 10 g), Glycyrrhiza uralensis Fisch. ex DC. (Fabaceae, 6 g), Angelica sinensis (Oliv.) Diels (Apiaceae, 10 g), Citrus reticulata Blanco (Rutaceae, 6 g), Actaea cimicifuga L. (Ranunculaceae, 3 g), Bupleurum chinense DC. (Apiaceae, 3 g) (in accordance with the original formula ratio in *Treatise on the Spleen and Stomach* by Li Gao).

Buzhong Yiqi Decoction was first recorded in *Li Gao’s Differentiation of Internal* and *External Injuries: Discussion on Diet and Fatigue*, and was further elaborated in his *Treatise on the Spleen and Stomach: Discussion on the Adjustment of Diet and Temperature*. It was clearly indicated that the formula is used to treat “*disorders caused by improper diet and inappropriate temperature, which injure the spleen and stomach*”. The theoretical basis of the formula can be traced back to *Huangdi Neijing (Yellow Emperor’s Inner Classic)*: *Suwen (Essential Questions)*, which states: “For exhaustion, warm it; for damage, warm it. Warmness can dispel extreme heat. It is highly undesirable to use bitter and cold drugs that deplete the stomach.” The name “Buzhong Yiqi” specifically refers to tonifying the spleen and stomach in the middle-jiao, and replenishing the qi that governs the ascending and descending functions of the spleen and stomach. The spleen and stomach, located in the middle-jiao, are the acquired foundation of the human body. They are not only the fundamental source of qi and blood production to nourish the five zang organs, limbs and bones, but also the core pivot of qi movement in the whole body. Only when the spleen’s function of ascending clear qi and the stomach’s function of descending turbid qi are coordinated, can the spleen and stomach generate qi normally. If the spleen and stomach are damaged, it will lead to middle-jiao qi deficiency and qi sinking. In this condition, the clear yang qi of the spleen cannot ascend, and the turbid yin qi of the stomach cannot descend, resulting in impaired transformation of water and grain and failure of essence and qi generation. This will damage the body’s vital energy and cause various diseases. Patients with middle-jiao qi deficiency and sinking syndrome often present with sallow or pale puffy complexion, shortness of breath, reluctance to speak, listlessness, weak limbs, loose stools, and excessive sweating. Some may experience bleeding or vaginal discharge, occasional fever and headache with a preference for warm drinks, and a pale tongue with thin white coating. Therefore, Buzhong Yiqi Decoction exerts its therapeutic effect by replenishing middle-jiao qi, restoring the ascending and descending functions of the spleen and stomach, harmonizing the receiving and transforming functions of the spleen and stomach, and promoting the recovery of the body.

Modern studies have confirmed the anti-exercise-induced fatigue (anti-EIF) effect of Buzhong Yiqi Decoction. Peng et al. (Zhang, 2022) applied Buzhong Yiqi Decoction to a mouse model of swimming exhaustion, and found that the exhaustive swimming time of the treatment group was significantly prolonged, and the protein expression levels of Adipor1, phosphorylated Adenosine 5′-monophosphate-activated protein kinase/total Adenosine 5′-monophosphate-activated protein kinase (p-AMPK/AMPK), Peroxisome Proliferator-Activated Receptor γ Coactivator 1α (PGC-1α), and Hexokinase 2 (HK2) in skeletal muscle were significantly upregulated. In TCM theory, the core pathogenesis of middle-jiao qi deficiency and sinking syndrome is the dysfunction of the spleen and stomach in transporting and transforming nutrients, which leads to insufficient qi and blood production and inadequate energy supply to skeletal muscle. The Adipor1/AMPK/PGC-1α pathway is a key pathway regulating skeletal muscle fat metabolism and mitochondrial biogenesis. Activation of this pathway by the formula improves fat utilization efficiency, which is the modern molecular embodiment of the TCM therapeutic principle of “tonifying the middle-jiao and replenishing qi to nourish the muscles”. Wei et al. (2025) further found that Buzhong Yiqi Decoction exerts anti-EIF effect by regulating skeletal muscle mitochondrial homeostasis through the PTEN Induced Kinase 1 (PINK1)-centered signaling pathways, including Hypoxia-Inducible Factor 1α (HIF-1α)/PINK1/Parkin and HIF-1α/Hairy/Enhancer of Split Related with YRPW Motif 1 (HEY1)/PINK1 pathways. Mitochondria are the core site of cellular energy metabolism, and the imbalance of skeletal muscle mitochondrial homeostasis is a typical cellular manifestation of middle-jiao qi deficiency and sinking syndrome in TCM. The regulation of mitochondrial autophagy and homeostasis by the formula through the PINK1 pathway constitutes a molecular-level verification of the TCM theory of “invigorating the spleen and replenishing qi to nourish the pectoral qi as well as the muscles and tendons”. Yang and Qiao (2017) conducted a clinical study including 64 patients with exercise-induced fatigue, and found that the total effective rate of the observation group treated with Buzhong Yiqi Decoction was as high as 96.87%, which was significantly higher than that of the control group. After treatment, the serum IgM and IgA levels of the observation group were significantly higher than those of the control group, suggesting that Buzhong Yiqi Decoction can effectively enhance the immune function of patients and promote physical recovery. This formula is indicated for middle-jiao qi deficiency and sinking syndrome, which corresponds to skeletal muscle energy metabolism disorder in EIF. Its modern mechanism of regulating the Adipor1/AMPK/PGC-1α pathway is highly consistent with the TCM theory of “tonifying the middle-jiao and replenishing qi to consolidate the muscles and interstitial spaces”.

### 5.2 Sijunzi Decoction

Core ingredients and classic proportion: Panax ginseng C. A. Mey. (Araliaceae, 10 g), Atractylodes macrocephala Koidz. (Asteraceae, 10 g), Poria cocos (Schw.) Wolf (Polyporaceae, 10 g), Glycyrrhiza uralensis Fisch. ex DC. (Fabaceae, 6 g, stir-baked) (in accordance with the original formula ratio in *Taiping Huimin Heji Jufang (Imperial Grace Formulary of the Taiping Era)* of the Song Dynasty).

Sijunzi Decoction was first recorded in the Song Dynasty medical classic *Taiping Huimin Heji Jufang*, which clearly states that its efficacy is “to treat qi deficiency of defensive and nutritive qi, weakness of zang-fu organs, abdominal distension and fullness, borborygmus and diarrhea, vomiting and retching, and is highly suitable for regular consumption”. *Jingyue Quanshu (Complete Works of Jingyue)* proposed that “among external injuries to the spleen and stomach, overwork is the most damaging to the spleen. When the spleen is injured, the exterior and interior are interconnected, and the stomach is greatly affected”. *Yujimiyi* also mentioned that “overwork injures the spleen”, and pointed out that exercise training-induced fatigue is closely related to spleen qi deficiency and impaired transportation and transformation functions of the spleen and stomach. The combination of *Panax ginseng, Atractylodes macrocephala, Poria cocos and fried Glycyrrhiza uralensis* in this formula works synergistically to tonify spleen qi and harmonize the spleen and stomach, and is mainly indicated for spleen qi deficiency syndrome. In clinical practice, it is widely used to improve symptoms such as shortness of breath, fatigue, poor appetite, loose stools, pale complexion and low voice caused by spleen qi deficiency, with significant therapeutic effects.

Modern studies have confirmed the anti-exercise-induced fatigue (anti-EIF) effect of Sijunzi Decoction. Hao (2014) found through animal experiments that Sijunzi Decoction could enhance the activity of Lactate Dehydrogenase (LDH) in rats, increase liver and muscle glycogen reserves, and reduce blood lactate levels, thereby prolonging the exhaustive exercise time of rats. In TCM theory, the core feature of spleen qi deficiency is impaired transportation and transformation function, which leads to insufficient production and storage of nutrients in the body. The reduction of liver and muscle glycogen is the material manifestation of this pathological change in the energy metabolism system. The formula increases glycogen reserves and enhances LDH activity to accelerate lactate metabolism, which essentially realizes the TCM therapeutic effect of “invigorating the spleen and replenishing qi to generate qi and blood for energy supply”. Pang et al. (2024) found that Sijunzi Decoction significantly regulated the Adenosine 5′-monophosphate-activated protein kinase/Sirtuin 1 (AMPK/SIRT1) signaling pathway in mice with exercise-induced fatigue. The results showed that the protein expression levels of AMPK and SIRT1 in the high and medium-dose groups were significantly higher than those in the low-dose group (P < 0.05). The AMPK/SIRT1 pathway is a key regulator of cellular energy metabolism and oxidative stress homeostasis, and decreased activity of this pathway is the molecular basis for persistent fatigue caused by spleen qi deficiency. The formula modulates the activity of this pathway to restore the body’s energy metabolic balance, which is a modern molecular interpretation of the TCM theory of “invigorating the spleen and replenishing qi to relieve fatigue”. This confirms that the formula can effectively alleviate spleen deficiency symptoms in mice with exercise-induced fatigue by regulating AMPK/SIRT1 activity. Pan and Zhang (2024) conducted a randomized controlled trial in patients with chronic fatigue syndrome of spleen deficiency type, and found that Sijunzi Decoction could significantly improve sleep disorders, relieve tension and anxiety, enhance memory, reduce fatigue scores, improve oxidative stress status, and elevate the quality of life of patients., This formula is indicated for spleen qi deficiency syndrome, and improves energy metabolism by activating the AMPK/SIRT1 pathway, which is highly consistent with the core TCM theory that “the spleen is the acquired foundation and the source of qi and blood production”.

### 5.3 Shengmai Decoction

Core ingredients and classic proportion: Panax ginseng C. A. Mey. (Araliaceae, 10 g), Ophiopogon japonicus (Thunb.) Ker Gawl. (Asparagaceae, 15 g), Schisandra chinensis (Turcz.) Baill. (Schisandraceae, 6 g) (in accordance with the original formula ratio in *Medical Origin* by Zhang Yuansu).

Shengmai Decoction originated from Zhang Yuansu’s Medical Origin in the Jin Dynasty, which records that it can “treat qi and blood deficiency, weakness of internal organs, abdominal distension and fullness, borborygmus and diarrhea, vomiting and retching, and is highly recommended for clinical use”. The “*Pharmacopoeia of the People’s Republic of China*” clearly specifies that its core therapeutic effects are “tonifying qi and restoring the pulse, nourishing yin and generating body fluids; indicated for qi and yin deficiency, palpitations, shortness of breath, weak pulse, and spontaneous sweating”. This formula is composed of three botanical drugs: Panax ginseng, Ophiopogon japonicus and Schisandra chinensis, with the core efficacy of tonifying qi, nourishing yin, and nourishing the heart and lungs. From the perspective of formula compatibility logic, on the one hand, it tonifies qi and strengthens the spleen to nourish the acquired foundation, ensuring the production of qi and blood, and improving symptoms related to spleen qi deficiency; on the other hand, it nourishes yin and generates body fluids to nourish the heart and lungs, effectively alleviating discomfort caused by qi and yin deficiency. In clinical practice, it is commonly used to treat symptoms such as shortness of breath, fatigue, palpitations, spontaneous sweating, and general weakness related to qi and yin deficiency syndrome.

Animal experiments have shown that Shengmai Decoction and its modified preparations can enhance the body’s antioxidant capacity and promote glycogen synthesis to exert anti-fatigue effects. Among them, Wujia Shengmai Decoction has a more prominent anti-fatigue effect, and its mechanism is related to the upregulation of protein expressions including phosphorylated phosphatidylinositol 3-kinase (p-PI3K) and phosphorylated protein kinase B (p-AKT) (Han et al., 2024). In TCM theory, qi and yin deficiency syndrome is characterized by qi consumption and yin impairment, which leads to insufficient energy supply and excessive oxidative stress in the body. The PI3K/AKT pathway is closely related to the regulation of cellular energy metabolism and antioxidant capacity. The formula upregulates the expression of p-PI3K and p-AKT to enhance antioxidant capacity and promote glycogen synthesis, which essentially realizes the TCM therapeutic effect of tonifying qi and nourishing yin to relieve fatigue. Clinical studies have further verified its application value. Zou et al. (2012) conducted a randomized controlled trial involving 14 female taekwondo athletes, and found that Shengmai Decoction not only improved the athletes’ aerobic exercise capacity, but also increased immunoglobulin levels, thereby enhancing the body’s immune function to a certain extent. Athletes under long-term high-intensity training are prone to qi and yin deficiency due to excessive consumption of qi and body fluids, which manifests as post-exercise fatigue, spontaneous sweating and decreased exercise capacity. The improvement of aerobic capacity and immune function by Shengmai Decoction is a specific embodiment of its TCM effect of tonifying qi and nourishing yin to replenish consumed qi and body fluids. This formula is indicated for qi and yin deficiency syndrome. It replenishes energy and enhances antioxidant capacity through the PI3K/AKT pathway, which precisely corresponds to the TCM efficacy of “tonifying qi and nourishing yin, promoting the production of body fluids to stop sweating”.

### 5.4 Xiaochaihu Decoction

Core ingredients and classic proportion: Panax ginseng C. A. Mey. (Araliaceae, 10 g), Bupleurum chinense DC. (Apiaceae, 12 g), Scutellaria baicalensis Georgi (Lamiaceae, 9 g), Pinellia ternata (Thunb.) Makino (Araceae, 9 g), Glycyrrhiza uralensis Fisch. ex DC. (Fabaceae, 6 g), Zingiber officinale Roscoe (Zingiberaceae, 3 slices), Ziziphus jujuba Mill. (Rhamnaceae, 5 pieces) (in accordance with the original formula ratio in *Treatise on Cold Damage Disorders* by Zhang Zhongjing).

The clinical application basis of Xiaochaihu Decoction is derived from *Treatise on Cold Damage Disorders*. Article 96 of the book clearly records that “after five or 6 days of cold damage or wind injury, alternating chills and fever, fullness and pain in the chest and hypochondrium, listlessness, loss of appetite, restlessness, and frequent vomiting… Xiaochaihu Decoction should be prescribed.” From the perspective of its efficacy mechanism, Xiaochaihu Decoction exerts the effects of harmonizing Shaoyang, tonifying the middle-jiao, and reinforcing healthy qi by regulating the qi movement of the three jiao, harmonizing the functions of upper and lower viscera, and promoting the communication between the exterior and interior of the body. In the treatment of deficiency disorders, it harmonizes the Shaoyang pivot, relieves qi movement stagnation, and reinforces healthy qi while eliminating pathogenic factors. Thus, it can effectively alleviate patients’ discomfort such as hypochondriac fullness and pain, restlessness, bitter taste in the mouth, dry throat, and dizziness, promote the smooth flow of qi, realize the internal and external harmony of viscera and the balance of yin and yang, and thereby relieve fatigue symptoms.

Relevant studies have confirmed the anti-fatigue efficacy of Xiaochaihu Decoction. Sheng et al. (2022) found that Xiaochaihu Decoction could effectively reduce the Symptom Checklist 90 (SCL-90) scores of patients, with a significant alleviation effect on fatigue. In TCM theory, Shaoyang disharmony and qi stagnation syndrome often leads to blocked qi movement, which further causes mental restlessness, listlessness and persistent fatigue. The reduction of SCL-90 scores reflects the improvement of these fatigue-related symptoms by Xiaochaihu Decoction, which is essentially the clinical manifestation of its efficacy in harmonizing Shaoyang and smoothing qi movement. Guo (2023) conducted an intervention experiment on exercise-induced fatigue mice with Xiaochaihu Decoction. The results showed that the endurance and muscle strength of the treated mice were significantly improved, and the serum levels of fatigue-related indicators including Creatine Kinase (CK), Blood Urea Nitrogen (BUN), and Lactate Dehydrogenase (LDH) were decreased. In addition, the levels of antioxidant indicators including Catalase (CAT), Superoxide Dismutase (SOD), and Glutathione (GSH) in the body were higher than those in the control group, suggesting that both the anti-fatigue and antioxidant capabilities of the mice were enhanced. n TCM theory, Shaoyang disharmony and qi stagnation can lead to disordered qi and blood circulation, abnormal body metabolism, accumulation of metabolic wastes (such as urea nitrogen) and excessive free radical production, which further aggravate exercise-induced fatigue. Xiaochaihu Decoction reduces the levels of CK, BUN and LDH to alleviate metabolic disorders, and increases the contents of SOD, CAT and GSH to enhance antioxidant capacity, which is the material basis for its TCM efficacy of harmonizing Shaoyang, smoothing qi movement and relieving fatigue. A randomized clinical trial by Jiang (2023) found that patients treated with Buzhong Yiqi Decoction combined with Xiaochaihu Decoction showed more significant improvements in overall fatigue, physical fatigue, mental fatigue, vitality reduction, and lack of motivation, compared with the control group treated with adenosine triphosphate tablets alone. Clinically, patients with exercise-induced fatigue often have concurrent Shaoyang disharmony and middle-jiao qi deficiency. Xiaochaihu Decoction focuses on harmonizing Shaoyang and smoothing qi movement, while Buzhong Yiqi Decoction focuses on tonifying middle-jiao qi, and their combination exerts complementary anti-fatigue effects. This further verifies that the anti-fatigue effect of Xiaochaihu Decoction is closely related to its TCM efficacy of harmonizing Shaoyang and regulating qi movement. This formula is indicated for Shaoyang disharmony and qi stagnation syndrome in TCM. Its effects of reducing oxidative stress and regulating energy metabolism indicators in EIF are highly consistent with the TCM therapeutic principle of “harmonizing Shaoyang and smoothing qi movement”.

## 6 Contemporary medical experts’ experience formulations

Liu W. et al. (2019) ice after a loaded swimming experiment, and found that “Fresh Ginseng Paste”, a processed product of ginseng, could significantly prolong the loaded swimming time of mice, effectively reduce the serum levels of urea nitrogen and lactic acid in mice after exercise, and markedly increase liver glycogen reserves. This study initially confirmed that Fresh Ginseng Paste has a significant alleviating effect on exercise-induced fatigue (EIF). Wang et al. (2010a), Wang et al. (2010b), and Wang et al. (2010c) found through animal experiments that Liqi Tiaobu Decoction could regulate the adaptability of the Hypothalamic-Pituitary-Adrenal (HPA) axis in fatigued rats, reduce excessive serum cortisol release, enhance T cell function, and promote cytokine secretion to maintain immune homeostasis. It can also achieve balanced regulation of the neuroendocrine-immune network by modulating the levels of serum β-Endorphin (β-EP) and Interleukin 2 (IL-2), maintain body system stability, and improve the exercise capacity of fatigued rats. Wang (2023) conducted a study on the effect of Shenjiang Decoction on EIF model mice, and found that Shenjiang Decoction could not only prolong the swimming time of fatigued mice and improve their fatigue state, but also reduce muscle fiber and mitochondrial damage, enhance antioxidant capacity, and maintain energy metabolism homeostasis. The formula exerts anti-EIF effects by regulating autophagy and the Adenosine 5′-monophosphate-activated protein kinase/Peroxisome Proliferator-Activated Receptor γ Coactivator 1α (AMPK/PGC-1α) signaling pathway. Inhibition of AMPK/PGC-1α pathway activity will lead to abnormal changes in the autophagy process. Based on this, it is speculated that Shenjiang Decoction regulates the autophagy mechanism by activating the AMPK/PGC-1α signaling pathway, and ultimately exerts anti-fatigue effects. Wang et al. (2024) found through network pharmacology that the sovereign, minister, and assistant drugs in Shengjing Buxue Decoction have differential and complementary effects. These components not only act on EIF-related pathways including the IL-17 signaling pathway, PI3K/Akt signaling pathway, and Th17 cell differentiation, but also jointly target the Janus Kinase-Signal Transducer and Activator of Transcription (JAK-STAT) signaling pathway to maintain the normal function of skeletal muscle and exert anti-EIF effects.

At present, research on contemporary ginseng-containing empirical formulas is mostly limited to small-sample animal experiments (n < 50) or single-center clinical observations (n < 100), lacking well-designed multicenter double-blind randomized controlled trials (RCTs). Most outcome measures are based on subjective fatigue scores, while the detection of objective indicators (e.g., mitochondrial function, short-chain fatty acid (SCFA) levels) is insufficient. Therefore, the rigor of clinical verification for these formulas needs to be further strengthened in future studies (Table 1).

**TABLE 1:** Core information summary: ginseng-containing classical and contemporary formulas for EIF prevention and treatment.

Formula category	Formula name	Core TCM efficacy	Key botanical drug composition	Key research findings and mechanisms
Classical ginseng formulas	Buzhong Yiqi Decoction	Tonify middle-Jiao qi, restore spleen-stomach func.	Astragalus membranaceus, Panax ginseng, Atractylodes macrocephala, etc.	Mice exhaustion time↑, 96.87% clinical efficacy, IgM/IgA↑; regulated adipor1/AMPK/PGC-1α pathways
	Sijunzi Decoction	Invigorate spleen qi, harmonize spleen-stomach	Panax ginseng, Atractylodes macrocephala, Poria cocos, Glycyrrhiza uralensis (stir-baked)	Glycogen reserves↑, blood lac/fatigue scores↓; modulated energy metabolism via AMPK/SIRT1 pathway
	Shengmai Decoction	Replenish qi, nourish yin, promote fluid	Panax ginseng, Ophiopogon japonicus, Schisandra chinensis	Antiox capacity↑, athletes’ aerobic cap/Ig↑; anti-fatigue via PI3K-Akt pathway
	Xiaochaihu Decoction	Harmonize Shaoyang, tonify healthy qi	Bupleurum chinense, Scutellaria baicalensis, Pinellia ternata, etc.	Mice endurance/antiox capacity↑, SCL-90 scores/fatigue indices↓
Contemporary empirical formulas	Fresh Ginseng Ointment	Tonify qi, relieve fatigue	Processed fresh ginseng	Mice loaded swimming time/liver glycogen↑, post-exercise BUN/Lac↓
	Liqi Tiaobu Decoction	Regulate qi, tonify deficiency, balance NEI network	Qi-regulating and tonic botanical drugs (unspecified)	Regulated HPA axis, T cell function↑; balanced NEI network via β-EP/IL-2
	Shenjiang Decoction	Tonify qi, warm yang, relieve fatigue	Ginseng, Zingiberis Recens	Swimming time/antiox capacity↑, myofiber/mitochondrial damage↓; activated AMPK/PGC-1α pathway
	Shengjing Buxue Decoction	Generate essence, tonify blood, relieve fatigue	Monarch-minister-assistant-guider botanical drugs (unspecified)	Targeted IL-17/PI3K-Akt/JAK-STAT pathways, maintained skeletal muscle func

1. Compiled from the original article’s animal/clinical/network pharmacology studies, aiming to present differences in TCM efficacy, composition, and anti-fatigue mechanisms of ginseng-containing formulas; 2. Key abbreviations: BUN (Blood Urea Nitrogen), AMPK, PGC-1α, SIRT1, PI3K-Akt, HPA, axis (Hypothalamic-Pituitary-Adrenal Axis), β-EP (β-Endorphin), IL-2, SCL-90, IgM, IgA; 3. “Unspecified” in “Key botanical drug Composition” indicates unclear specific herbal ingredients in the original article; 4. Clinical effective rate and indicator changes are directly quoted from the original article (see references therein).

## 7 Conclusion

Ginseng and its related ginseng-containing formulas exert anti-exercise-induced fatigue (EIF) effects through multiple pathways. These include regulating the binding of cytokines and cell adhesion molecules, modulating tumor necrosis factor expression, and targeting glucose metabolism disorders and lactic acid accumulation to alleviate peripheral fatigue. The clinical application of these formulas requires syndrome differentiation-based treatment to achieve precise medication. This review systematically consolidates the research progress of ginseng and ginseng-containing formulas in the prevention and treatment of EIF, sorts out the complementary and potential synergistic anti-fatigue effects of their multiple active metabolites, and summarizes the efficacy of classic and contemporary empirical formulas. It provides a comprehensive reference for clinical practice, and clarifies the directions for subsequent research. At present, the screening of core active metabolites and the analysis of multi-target regulatory mechanisms are still in the initial stage, and lack the support of systematic clinical translational research. Future research should be based on the holistic characteristics of traditional Chinese medicine (TCM), and integrate modern medical technologies and interdisciplinary research approaches. For mechanistic research, it is recommended to prioritize the validation of three key pathways: AMPK/PGC-1α (energy metabolism), PINK1/Parkin (mitochondrial autophagy), and gut microbiota-short chain fatty acid (SCFA) (metabolic mediation). These are the core targets of ginseng against EIF, while the existing evidence is fragmented and requires further verification through human experiments. Future research should focus on three key directions: first, conducting human association studies to confirm the mediating role of Bifidobacterium and *Lactobacillus* in the anti-EIF effect of ginseng; second, designing large-sample, multicenter randomized controlled trials with objective exercise indicators as primary outcomes; third, standardizing the content of active metabolites in ginseng extracts, and formulating unified quality control standards for ginseng-containing formulas. Integrating TCM prescription compatibility rules and syndrome differentiation-based medication characteristics will further promote the innovative development of TCM-based precise treatment for EIF, and provide solid theoretical and practical support for clinical practice.
